# The emergent property of inhibitory control: implications of intermittent network-based fNIRS neurofeedback training

**DOI:** 10.3389/fnhum.2025.1513304

**Published:** 2025-03-04

**Authors:** Lingwei Zeng, Lidong Gai, Kewei Sun, Yimeng Yuan, Yuntao Gao, Hui Wang, Xiucao Wang, Zhihong Wen

**Affiliations:** ^1^Military Medical Psychology School, Fourth Military Medical University, Xi’an, China; ^2^The First Regiment of the Basic Training Base of the Air Force Aviation University, Changchun, China; ^3^Department of Aerospace Medicine, Fourth Military Medical University, Xi’an, China

**Keywords:** functional near-infrared spectroscopy, neurofeedback training, emergent property, inhibitory control, small-world network

## Abstract

**Background:**

Studies have shown that inhibitory control is supported by frontal cortex and small-world brain networks. However, it remains unclear how regulating the topology changes the inhibitory control. We investigated the effects of small-worldness upregulation training on resting-state networks via fNIRS neurofeedback training, which will contribute to a deeper insight of inhibitory control.

**Methods:**

A five-day training session was used to regulate the small-worldness of the frontal cortex, and the color-word Stroop task was tested before and after training. Fifty healthy adults were recruited and randomly assigned to the sham feedback group (sham group), or intermittent fNIRS-based brain network feedback group (fNIRS-NF group). On the basis of the exclusion of incomplete data, 45 valid data sets were retained and analyzed (sham: 21, fNIRS-NF: 24).

**Results:**

Training increased resting-state small-worldness and improved Stroop task performance, with a significant correlation between these changes (*r* = −0.32, *p* = 0.032). The fNIRS-NF group exhibited reduced hemodynamic activation (βvalue decreased, indicating lower cognitive load) during posttest and follow-up. Notably, the right dorsolateral prefrontal cortex (dlPFC) showed greater intra-regional connectivity increases than the left dlPFC, suggesting asymmetric plasticity.

**Conclusion:**

Intermittent fNIRS neurofeedback effectively modulates resting-state small-world networks and enhances inhibitory control, with effects sustained for at least one week. These findings highlight small-worldness as a novel target for cognitive interventions.

## Graphical Abstract

Based on the small-world network model, intermittent neurofeedback training can directly regulate resting-state brain network properties. This intervention not only enhanced small-world characteristics but also coincided with reduced conflict effects in the Stroop task. Correlation analyses revealed a causal relationship between small-world network properties and inhibitory control. Notably, participants receiving real feedback demonstrated significantly lower brain activation during post-training Stroop task performance compared to the sham feedback group, indicating reduced cognitive load. Although the feedback algorithm exhibited no regional selectivity, we observed preferential increases in right dorsolateral prefrontal cortex Based on the small-world network model, intermittent neurofeedback training directly modulates resting-state brain network. This intervention enhanced small-world properties while simultaneously reducing the conflict effect in the Stroop task. Correlational analyses further revealed a causal link between small-world network property and inhibitory control. Additionally, we observed that participants in the fNIRS-NF group exhibited reduced brain activation during the same Stroop task post-training, indicating lower cognitive load compared to the sham feedback group. Although the feedback algorithm did not selectively target specific brain regions (nodes), the fNIRS-NF group showed a greater increase in functional connections within the right dorsolateral prefrontal cortex (DLPFC) compared to the left DLPFC. This asymmetry may reflect inherent differences in neuroplasticity across brain regions. 
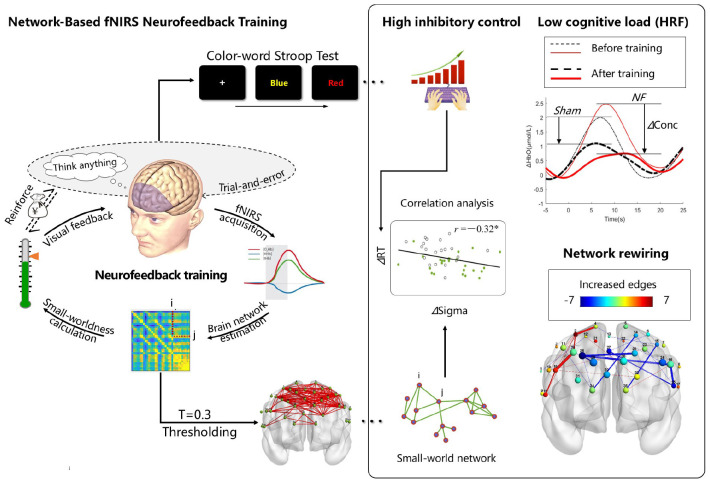

## Introduction

Application of graph theory to noninvasive brain imaging in humans has yielded critical insight into mechanisms of cognitive control (Bassett and Khambhati, 2017). Could a subject be trained to modulate the graph property of the global network? Previous studies have shown that can be achieved by fNIRS neurofeedback training in the training state (Zeng et al., 2023), but whether the resting state network can be modulated is still unknown.

In neuroscience, there is a growing consensus that functions form an emerging property of the interactions between brain areas (Pessoa, 2022). Emergence refers to the unexpected collective spatiotemporal patterns exhibited by large complex systems, which are composed of many nonlinear interacting elements (Chialvo, 2010). For example, facial recognition and working memory are emergent functions that combine many small pieces to create a greater whole (de Haan et al., 2002; Hernandez et al., 2019; Miller et al., 2024; Postle, 2006), whereas the linguistic form emerges from the operation of self-organizing systems (D’Souza and D’Souza, 2019; Leary, 1999; Macwhinney, 1998). Anderson proposed that higher cognitive functions can reuse brain regions that emerged earlier in the evolutionary process and thus yield more scattered activations. He also asserted that areas at the front of the brain are evolutionarily newer, whereas those at the back of the brain are older (Anderson, 2016; Anderson and Penner-Wilger, 2013). Inhibitory control is an advanced cognitive function that is closely related to the prefrontal cortex (PFC), but much of our current knowledge of the role of the PFC in cognitive control has been derived from the modular paradigm, in which specific functions are ascribed to localized subdivisions of the PFC, with the underlying assumption being that they act as independent processors for specific cognitive functions (Menon and D’Esposito, 2022).

Response inhibition paradigms provide important markers for clinical research and assessments. However, the inhibitory control hypothesis is controversial. Several researchers have argued that the attempt to map a discrete inhibitory ability onto a dedicated brain region is misguided (Erika-Florence et al., 2014; Hampshire and Sharp, 2015). Hampshire proposed the idea that inhibitory control is an emergent property of biased local competition and that behavioral inhibition is an emergent property of common neural mechanisms that are ubiquitous throughout systems in the human brain (Erika-Florence et al., 2014; Hampshire and Sharp, 2015). From a biological evolutionary perspective, these studies inferred that higher cognitive functions may be more involved in networked tendencies and tried to find evidence to support this.

In recent years, more graph theory methods have been used for fNIRS research, (Li et al., 2024). Researchers have theorized that the human brain has significant small-world properties, which are the result of natural selection of the brain under a cost–efficiency balance (Bassett and Bullmore, 2016; Meunier et al., 2010). Humphries defined a precise measure of “small-worldness” according to the trade-off between high clustering coefficient (Gamma) and short characteristic path length (Lambda), and small-worldness (Sigma = Gamma/Lambda) was derived from these metrics to determine a quantitative, continuous grading of network status (Humphries and Gurney, 2008). Several graph theory-based studies have shown that small-world property in the brain influence inhibitory control. For example, the core deficit in attention-deficit hyperactivity disorder (ADHD) patients is inhibitory control, and the brains of these patients exhibit regularized changes in small-world networks (Wang et al., 2009), whereas self-control behaviors related to inhibitory control show increased randomness in brain networks (Zhang et al., 2016). These studies attempted to support a network view of inhibitory control through correlation analysis. However, correlation is not a strong causal inference. To find conclusive evidence that inhibitory control is supported by the underlying network, we can perturb the brain network topology directionally through neurofeedback training.

Neurofeedback training is a promising approach for perturbing the brain network (Pamplona et al., 2020; Xia et al., 2021; Yu et al., 2021; Zeng et al., 2023), and it is one of the few methods that can directly modulate the brain network topology property. This method can answer the question of whether a causal relationship is present between the global topological properties of the network and the emergence of inhibitory control.

Resting-state functional connectivity could pinpoint network hubs, such as cognitive control areas (Cole et al., 2013), that interact with many other brain areas and serve as a potential target for modulating distributed brain dynamics with neurofeedback. It is intuitively resting-state brain network regulation is difficult because it lacks the effects of “stimulus-response automation” (Beauchamp et al., 2016); and relies only on some form of “muscle strength” exercise (Baumeister et al., 2007; Wang et al., 2023), but it may be helpful to embed the transfer task in the training. For the functional near-infrared spectroscopy (fNIRS) neurofeedback training, several studies have proposed that intermittent feedback is superior to continuous feedback (Emmert et al., 2017; Hellrung et al., 2018; Johnson et al., 2012; Oblak et al., 2017). This is due to (1) the inherent delays exhibited by hemodynamic responses and the related feedback and (2) the limited cognitive resources possessed by individuals (Hellrung et al., 2018); individuals cannot effectively handle feedback information and self-regulation tasks simultaneously, especially for the purpose of resting-state perturbation. While the stimulation of continuous feedback distracts the subject, no feedback trials are embedded as a transfer task in intermittent feedback training, which is closer to the real resting state.

In this study, small-worldness was selected for upregulation-based brain network training, where intermittent fNIRS neurofeedback was used to perturb resting-state brain networks, a color-word Stroop task (CWST) was used to evaluate inhibitory control performance, and a sham feedback group was recruited for control purposes.

## Materials and methods

### Trial settings and sample size

The trial was conducted in Xi’an, China, from November 2023 to January 2024. The study was approved by the Ethics Review Committee of the Fourth Military Medical University, and all the subjects carefully read and signed the informed consent form prior to the cognitive experiments. The experiment adopted a randomized, double-blind design.

The study consisted of cognitive performance measurements at three time points: before the training process (pretest), 1 day after the training process (posttest), and 1 week after the end of training (follow-up assessments).

The sample size was calculated via G*Power version 3.1, where the mixed-design repeated-measures analysis of variance (ANOVA) model was adapted. We expected a medium inhibitory control performance effect on the basis of a previous NF study that reported a medium-to-large effect on executive functions (Da Silva and De Souza, 2021). The effect size was set at 0.25, α = 0.05, 1-β = 0.8, and the number of measurements was 2. The required minimum sample size was 34 (17 participants in each group).

### Participants

A total of 50 healthy adults were recruited to participate in this study. The participants were all medical college students, and patients with brain trauma or mental illness and those who underwent recent cognitive experiments were excluded. The participants were randomly assigned to the sham feedback group (sham) or intermittent fNIRS neurofeedback group (fNIRS-NF). Their gender, age, and educational background were balanced, but the hormonal state of women of reproductive age was not taken into account, which may be an important influencing factor (Bazanova et al., 2017). The participants were not told what group they were in, nor was the experimenter, and they were all asked if they knew their experimental grouping after the experiment. All the subjects completed the Edinburgh inventory (Oldfield, 1971), and all of them were right-handed. The data from 4 subjects were excluded because of incomplete data records. The data from 21 subjects in the Sham group (14 females) and 24 subjects in the fNIRS-NF group (11 females) were used in this study. We used the independent *t*-test to compare the ages and education years of the groups, and no significant differences were observed, as shown in Table 1.

**TABLE 1:** Demographic information for the participants in each group.

	Number of participants	Age (SD) in years	Education years (SD)
Sham	21 (14 female)	21.42 (2.13)	15.82 (2.11)
fNIRS-NF	24 (11 female)	20.68 (1.35)	15.25 (1.32)
*p*-value	–	0.16	0.27

### Brain imaging tools and region of interest definition

**FIGURE 1:**
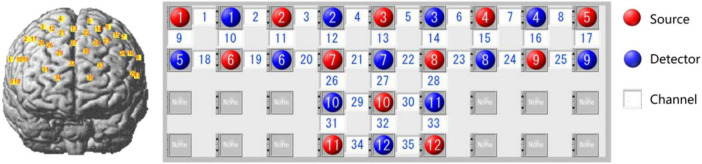
Schematic of channel layout (red dots, sources; blue dots, detectors; yellow or white squares, measurement channels; numbers indicate channel numbers).

In this study, an optical brain function imaging device (LABNIRS, Shimadzu Corp., Japan) was used to monitor the concentration of hemoglobin in the frontal cortex via a three-wavelength near-infrared semiconductor laser (780 nm, 805 nm, and 830 nm). The sampling rate was set to 25.6 Hz. The PFC was chosen as the ROI for feedback training because of its critical role in the IC. We designed a “T” layout on the frontal lobe, and the probe arrays allowed for 35 different measurement channels, with 3.0 cm of source-detector separation. Note that no short channel was employed in our experiment, as shown in Figure 1.

The actual coordinates of the channels were obtained via the 3D locator (Fastrak3d, Polhemus Corp., USA) from standard head mold, and then the mapping between the MNI coordinates and the Brodmann area was calculated via probability registration. The results showed that most channels are located in the traditionally defined PFC (BA8 to 14 and BA44 to 47) (Carlen, 2017), as shown in Table 2.

**TABLE 2:** Mapping of Brodmann area to channels.

Brodmann area	Right hemisphere	Left hemisphere
9(46)-Dorsolateral prefrontal cortex	1, 10, 18, 19, 29	16, 24, 25, 30
6-Premotor and supplementary motor cortex	3, 4, 12, 13	5, 6, 7, 8, 14
8- Includes frontal eye fields	2, 11, 20, 21, 26, 27	15, 22, 23, 28
45-Pars triangularis Broca’s area	9	17
10-Frontopolar area	31, 32, 34	33, 35

### fNIRS-NF protocols

**FIGURE 2:**
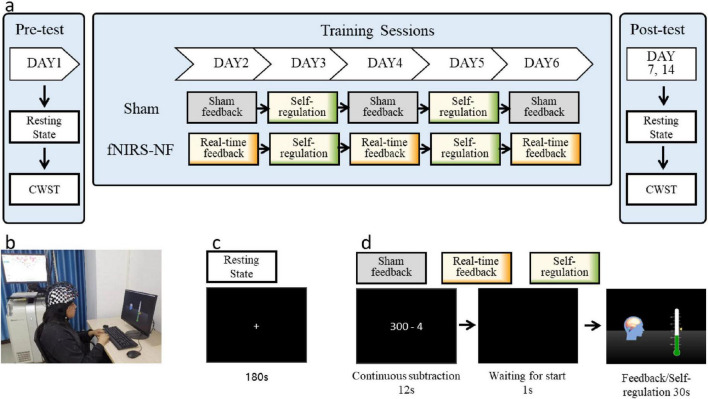
Experimental protocols. **(A)** The entire training and testing process of the experiment. **(B)** The fNIRS-NF training scenario. **(C)** The resting-state scan phase. “+” indicates fixation. **(D)** A single-block sequence of the training process. The sham feedback group was provided feedback signals recorded from other participants before; the real-time feedback block was provided true signals; and the self-regulation block did not receive feedback signals on days 3 and 5 but was provided their regulation scores after the daily session.

The neurofeedback training lasted for a total of 8 days, including behavioral tests on Days 1, 7, 14 and 5 training sessions from Days 2 to 6. There were 20 training blocks per day, with a 1-min rest at the end of every fifth block. The entire training protocol is shown in Figure 2A.

The participants sat in front of a computer with their eyes 60–70 cm away from the screen and kept their bodies, especially their heads, relaxed before starting the training process, as shown in Figure 2B. Before the everyday training process, a 180-s resting-state scan was performed by the subjects, who were asked to open their eyes and relax, keep their bodies and heads still, and try not to think about or recall anything, as shown in Figure 2C. The experimental instructions were presented after the resting-state scan, which required the participants to repeat two sequence tasks. In Task 1, participants were asked to count backward from a starting value using a specified increment for 12 s to limit their cognitive state as the baseline (Macduffie et al., 2018). In Task 2, one second after the black screen, the neurofeedback training regulation lasted for 30 s. The single-block task is shown in Figure 2D.

In the regulation phase, a green thermometer indicator bar and a yellow triangular slider were displayed on a black screen, where the triangle slider represented the immediate small-worldness of the brain network (for the calculation method, see the following section: Online Calculation of the Feedback Scores), whereas the thermometer indicator bar represented the cumulative small-worldness value of the brain network in the block (with a refresh rate of 3 Hz). The participants were instructed to increase the thermometer value as much as possible; they could use any psychological strategy other than breathing adjustments, physical changes, or facial expression movements; and they were informed that an additional monetary reward (up to CNY 900) would be paid in proportion with their increased scores. They received this money after all the experiments were completed.

### Behavioral testing

**FIGURE 3:**
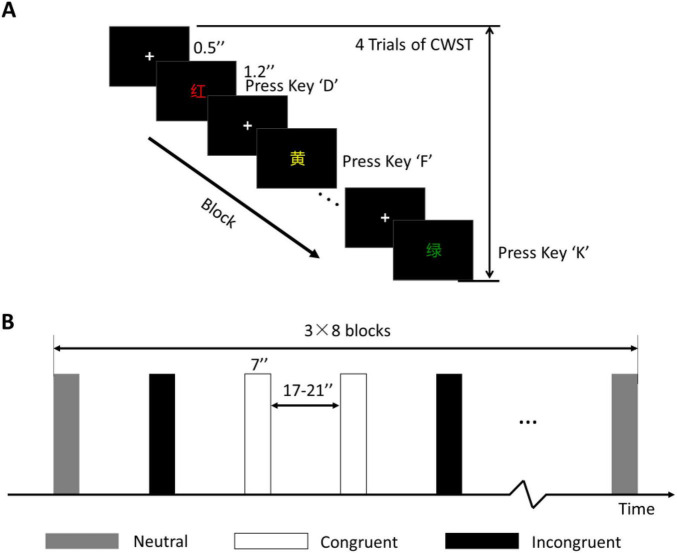
Behavioral test of the CWST. **(A)** One block of the CWST. **(B)** Block design and arrangement. Three conditions were included, and each condition consisted of 8 blocks with a counterbalanced order. “+” indicates fixation.

Because no significant difference in Stroop effect no matter whether Chinese or English orthographies were used (Lee and Chan, 2000), the Chinese version of color–word Stroop task (CWST, 4 colored Chinese characters) was used in this study as the behavioral test during the pretest, posttest, and follow-up test phases. During the experiment, one block consisted of 4 trials in sequence. A fixation was presented for 0.5 s, and each color-word trial was presented for 1.2 s. The participants rested for 17–21 s at the end of each block. A total of 24 blocks were pseudorandomly arranged in one task, and it took approximately 11 min, as shown in Figure 3.

### Online calculation of the feedback scores

We used the in-house MATLAB software R2017a (MathWorks Inc., Natick, Massachusetts, USA). This software runs on a connected computer and accesses data files in the NIRS system. The sampling rate of the employed fNIRS equipment was 25.6 Hz, and the segment length was 256 points; a time window of 10 s was provided to calculate the feedback score of the brain network. To reduce the physiological noise caused by heartbeats, respiration, and other physiological processes, the recorded fNIRS signals were filtered with a Butterworth bandpass filter with cutoff frequencies of 0.01 and 0.2 Hz. Finally, feedback latency was shown to be a critical parameter, and a shorter delayed latency is beneficial for imply learning (Belinskaya et al., 2020). Thus, the refresh rate was set to a maximum of 3 Hz (333 milliseconds feedback latency), which is limited by the computational performance.

The oxyhemoglobin (OxyHb) values of all the channels were recorded 10 s before, and the connection strength (*r*) between each channel pair was calculated via Pearson correlation. Then, the *r* values were converted into Z scores because they did not follow a Gaussian distribution. The conversion function was as follows:


$$Z=\frac{\text{ln}\,\left(1+r\right)}{2\,l\,n\,\left(1-r\right)}$$

**FIGURE 4:**
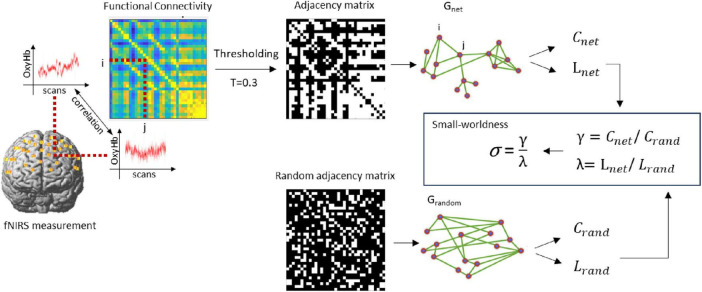
Schematic illustration of the small-worldness calculation. C_rand_ and L_rand_ were created by averaging the clustering-coefficient and path length of the random graphs. Next, γ and λ were computed, defined as C_net_/C_rand_ and L_net_/L_rand_, as well as the σ as the ratio between γ and λ expressing the small-worldness of G_net_.

As a result, a functional connectivity matrix M with a size of 35 × 35 was constructed, where the channels were nodes and the Z values were edges. Then, 30% of the larger edges are set to 1, whereas the other edges are set to 0 (an adjacency matrix with a sparsity of 0.3 is constructed). The small-worldness was calculated via the Humphries method via the Gretna v2.0.0 toolbox (Wang et al., 2015), as is shown in Figure 4.

The same method was used to calculate the baseline small-worldness sequence (the backward counting phase), and the feedback scores were corrected via the following formula (Xia et al., 2021):


$$S\,c\,o\,r\,e_{i}=\frac{50\,\left(\sigma_{i}-\sigma_{b\,a\,s\,e}+3\,S\,D\right)}{3\,S\,D}$$

Here, σ_*i*_ is the small-worldness of the *i*th trial, and σ_*base*_ and SD are the mean and standard deviation of the baseline small-worldness values, respectively. Feedback index was normalize to 1–100 through this transformation, when σ_*i*_ = σ_*base*_, score = 50; the scores were presented to the subjects and recorded in the hard disk, which were proportional to the participant’s monetary reward.

### Preprocessing and statistics of offline data

We performed the following preprocessing steps to offline data via NIRS_KIT v3.1 (Hou et al., 2021b) and Homer 2 (Huppert et al., 2009) based on MATLAB 2017a.

(1) The intensity data (raw data) were converted to optical density (OD) values.
(2) A bandpass filter (cutoff frequency: 0.01–1) is used to remove linear trends and artifacts preliminarily.
(3) Motion artifacts were identified on the basis of amplitude and standard deviation thresholds. If the signal for any channel changes by more than 40 times of standard deviation or 4 times of amplitude over 3 s interval, then this time point is marked as a motion artifact.
(4) Cubic spline correction was performed on the motion artifacts identified in step 3.
(5) A bandpass filter (cutoff frequency: 0.01–0.08) was applied to the data to further reduce the noise level (Pinti et al., 2019).
(6) The OD data were converted to concentrations. As young adult participants were included in our experiment, we chose [6.0 6.0 6.0] as the differential path length factor (DPF), as suggested in Chiarelli et al. (2019) study.

If it is task state (Stroop) data, two additional steps of processing were required:

(7) The denoised concentrations were segmented into blocks and averaged to the hemodynamic response function (HRF).
(8) HRFs were fitted by general linear model (GLM) to estimate activation quantity, named β.

To investigate whether the fNIRS-NF training group presented improved cognitive performance compared with the sham group, we analyzed the all-change Stroop effect via mixed-design repeated-measures ANOVA in SPSS 23. In addition, the relationships between improvements in inhibitory control (Stroop effect) and changes in the brain network (small-worldness) were investigated via Pearson correlation analysis. Finally, we analyzed the changing properties of node degrees, averaged and visualized the regulatory patterns of the brain network. In these analyses, *p* < 0.05 was considered significant, and multiple comparisons using false discovery rate (FDR) correction (Benjamini and Yekutieli, 2001).

## Results

### Changes in the feedback score

**FIGURE 5:**
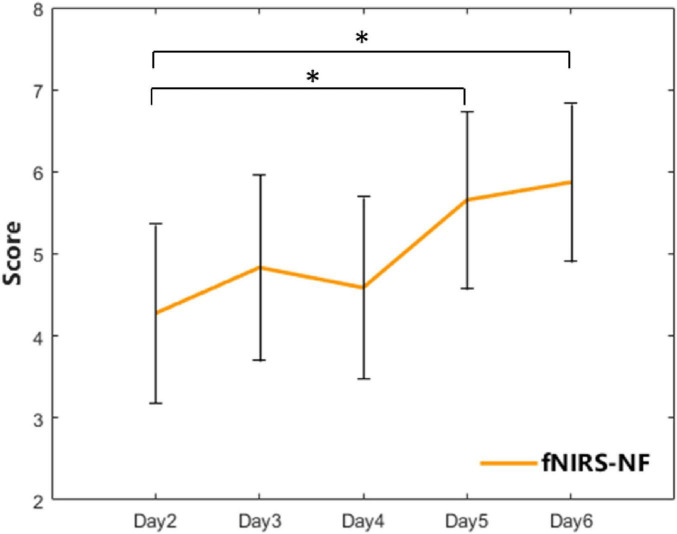
The feedback score of the fNIRS-NF group over 5 training sessions. The error bars indicate the SEM. A paired *t*-test was used for within-group analyses. *p < 0.05.

For the fNIRS-NF group, the feedback score showed a zigzag rise over 5 training sessions. The scores of the 4th and 5th training sessions significantly increased compared with those of the 1st training session (Day 4: *t* = −2.57, *p* = 0.017; Day 5: *t* = −2.36, *p* = 0.027), as shown in Figure 5.

### HRFs and brain activation of CWST

**FIGURE 6:**
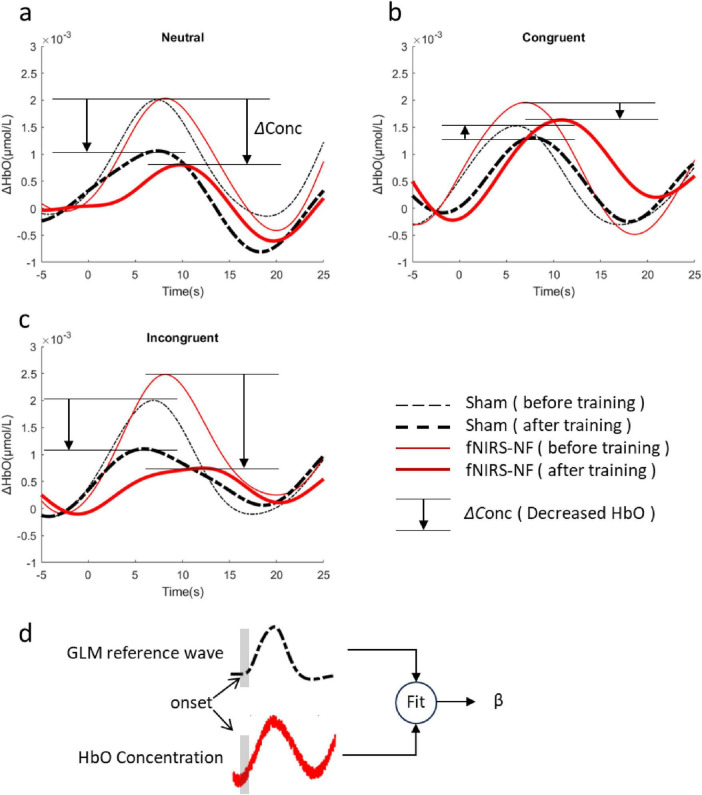
Changes in HRFs after training. **(A–C)** The grand average of all channels before (Day 1) and after (Day 7+Day 14) training for neutral congruent and incongruent condition. **(D)** Brain activation level (β) were estimated by GLM.

We calculated the average HRF across all channels for the CWST, and compared them before and after training. In general, the grand average OxyHb concentration of the fNIRS-NF group decreased more than that of sham group after training, especially for congruent and incongruent conditions, as shown in Figure 6. To further assess changes in brain activity of CWST, we estimated the level of brain activation using GLM, where OxyHb HRFs were fitted for β values. The β decrease during the test period was estimated and independent sample *t*-tests were performed, which showed that the fNIRS-NF group was significantly greater than the sham group during the posttest when the neutral condition and incongruent condition. The detailed statistical data were shown in Table 3. In addition, deoxyhemoglobin (DxyHb) showed a similar trend, which is not shown in this paper.

**TABLE 3:** Comparison between groups for the grand average of beta values.

Period	Condition	Group	Δβ (mean ± SDE, × 10^–4^)	*t*-value	*p*-value
Posttest-pretest	Neutral	Sham	−6.48 ± 2.64	2.27	0.028
		fNIR-NF	−15.07 ± 2.68		
	Congruent	Sham	−3.79 ± 2.02	1.65	0.106
		fNIR-NF	−10.24 ± 3.20		
	Incongruent	Sham	−10.30 ± 2.35	2.23	0.031
		fNIR-NF	−17.78 ± 2.38		
Follow-up-pretest	Neutral	Sham	−3.30 ± 1.97	0.16	0.874
		fNIR-NF	−3.82 ± 2.49		
	Congruent	Sham	−3.03 ± 2.44	1.81	0.077
		fNIR-NF	−8.95 ± 2.18		
	Incongruent	Sham	−7.01 ± 2.16	1.27	0.210
		fNIR-NF	−10.63 ± 1.87		

**FIGURE 7:**
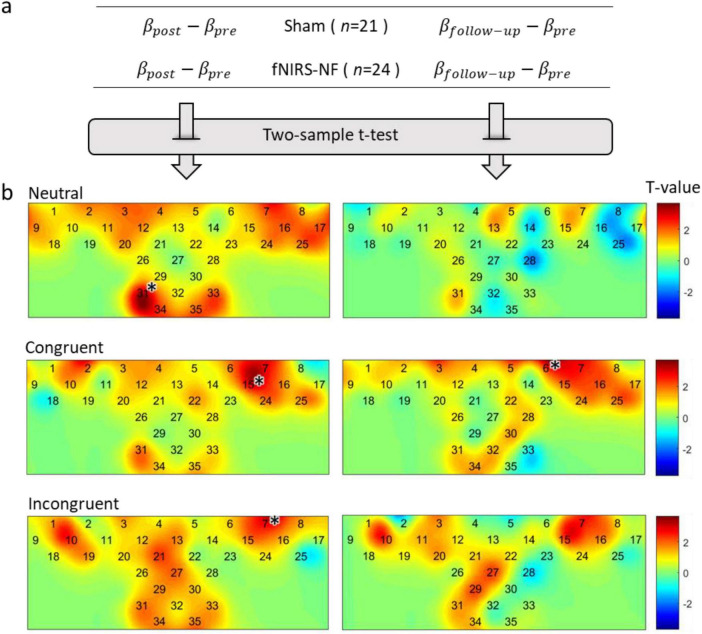
Changes in brain activation after training. **(A)** Δβ were calculated by subtracting the pretest from the posttest, and independent sample *t*-tests were performed. **(B)** In the figure below, the activation difference statistics (T-value) for each test period and each task condition were labeled on the channel layout. The level of significance was corrected by FDR (*p* < 0.05), *p < 0.05.

In addition, each channel was analyzed individually to identify key brain regions, results were shown in Figure 7.

The results of two sample *t*-test showed that the activation level decreased significantly in all task conditions during the post test period. This includes channel 31 for neutral condition (*t* = 3.76, *p* < 0.001), channel 15 for congruent condition (*t* = 3.41, *p* < 0.001), and channel 7 for incongruent condition (*t* = 3.42, *p* < 0.001). We further examined the long-term effect of training and found that the reduction of brain activation level could still be observed in the follow-up one week after the training. The results of two sample *t*-test showed that the activation level decreased significantly in congruent condition during the follow-up test in channel 6 (*t* = 3.61, *p* < 0.001).

### Changes in inhibitory control and small-worldness of resting state network

The Stroop effects were submitted to a mixed-design ANOVA. There was a significant main effect of day [*F*(1, 44) = 17.81, *p* = 0.000, η^2^ = 0.29], and group [*F*(1, 44) = 9.75, *p* = 0.003, partial η^2^ = 0.19]. The interaction effect between group and day was significant [*F*(1, 44) = 14.870, *p* = 0.000, partial η^2^ = 0.26]. Therefore, simple main effects were run. Stroop effect was not statistically significantly different in the fNIRS-NF group (M = 91.99, SEM = 8.51) compared to the sham group (M = 108.93, SEM = 9.09) at the beginning (pre-) of the training [*F*(1, 44) = 1.85, *p* = 0.181, partial η^2^ = 0.041]. However, Stroop effect resulted statistically significantly different in the fNIRS-NF group (M = 47.27, SEM = 9.83) compared to the Sham (M = 106.91, SEM = 10.51) at the end of the training (post-) [*F*(1, 44) = 17.19, *p* = 0.000, partial η^2^ = 0.286], a mean difference of =59.64, 95% CI [=88.65, =30.63]. Therefore, the pre–post comparison between the fNIRS-NF group and the sham group recorded a significant reduction in Stroop effect levels in the participants of the fNIRS-NF group.

The small-worldness were submitted to a mixed-design ANOVA. There was a significant main effect for group, but not for time. The small-worldness of the fNIRS-NF group was significantly greater than that of the sham group [*F*(1, 44) = 4.23, *p* = 0.046, partial η^2^ = 0.089]. The interaction effect between group and day was significant [*F*(1, 44) = 3.53, *p* = 0.034, partial η^2^ = 0.07]. Thus, simple effects were run. Small-worldness resulted significantly different in the fNIRS-NF group (M = 1.31, SEM = 0.03) compared to the Sham (M = 1.20, SEM = 0.03) at posttest [*F*(1, 44) = 6.19, *p* = 0.017, partial η^2^ = 0.13], 95% CI [−0.202, −0.021]. Therefore, the pre–post comparison between the fNIRS-NF group and the sham group recorded a significant reduction in small-worldness in the participants of the fNIRS-NF group.

**FIGURE 8:**
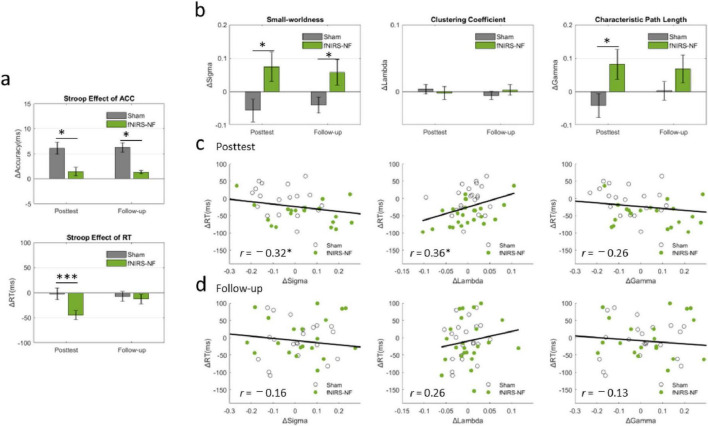
Changes in Inhibitory Control, Small-Worldness and their relationships. **(A)** Changes in the Stroop effect from the pretest data to the posttest (follow-up test) in the CWST. All the data are presented as the means (bars) ± standard errors (error bars). An independent-sample *t*-test was applied for between-group analyses. **(B)** Changes in small-worldness, clustering coefficient and characteristic path length during different periods after training. **(C,D)** Relationships between cognitive improvements and brain network changes. Pearson’s correlation was applied to analyze the cognitive-network correlations of all the subjects. *p < 0.05, ***p < 0.001.

We then took the pretest data as the baseline and subtracted them from the posttest (follow-up test) data to determine the lasting effects. Independent-sample *t*-tests between groups revealed that the decrease of Stroop effect at posttest in fNIRS-NF group was significantly greater than that in sham group (*t* = −3.86, *p* = 0.000), and the increase of small-worldness at posttest in fNIRS-NF group was significantly greater than that in sham group (*t* = −2.67, *p* = 0.011). And there was significant difference between groups at the follow-up test of small-worldness (*t* = −2.10, *p* = 0.043). We further extracted the clustering coefficient and characteristic path length in different periods, and the results showed that the characteristic path length of the fNIRS-NF group was significantly larger than that of the sham group (*t* = −2.11, *p* = 0.041). The results were shown in Figures 8A, B.

We then analyzed the correlation between the increment of resting-state small-worldness (ΔSigma) and cognitive improvement (ΔStroop) at posttest and follow-up tests. The results showed that ΔStroop was significantly negatively correlated with ΔSigma at posttest (*r* = −0.32, *p* = 0.032) and was significantly positively correlated with ΔLambda at posttest (*r* = 0.36, *p* = 0.014), as shown in Figures 8C, D.

### Differences in cortical plasticity

**FIGURE 9:**
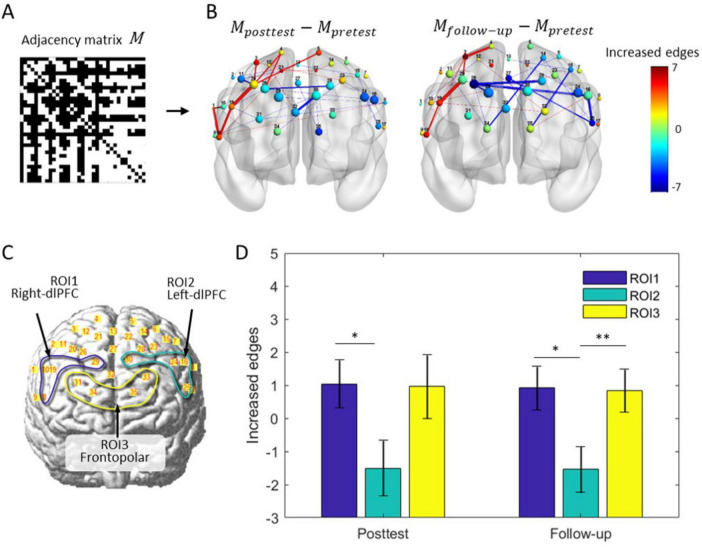
Differences in brain network rewiring and cortical plasticity. **(A)** The adjacency matrix of the brain network for the fNIRS-NF group. **(B)** Average changes in the brain network during training in the fNIRS-NF group. Thirty-five nodes are localized to the brain template according to their MNI coordinates, and their size indicates the nodal degree. The node color indicates the change in node degree from pretest to posttest (follow-up test). The color lines indicate the increase (red) or decrease (blue) in the edge, where the color bar indicates an increase (red end) or decrease (blue end) in the connections. **(C)** Definition of ROI1 (channel 8, 10, 19 and 29), ROI2 (channel 16, 24, 25 and 30) and ROI3 (channel 31, 33, 34 and 35). **(D)** Statistics of the number of connections within ROI. Paired *t*-test, *p < 0.05, **p < 0.01.

Due to the global topological properties of all nodes as modulation targets, the feedback algorithm has no selectivity for brain regions. However, the inherent plasticity differences in the cortex may lead to spatial imbalance in network evolution. We then extracted the adjacency matrix of the real group before and after training, obtained and visualized the differences in the brain network through subtraction, as is shown in Figures 9A, B. For cortical plasticity, BA9 and BA46 in the left hemisphere have a greater probability of decreasing connections, whereas marginal brain regions in the right hemisphere have a greater probability of increasing connections, as shown in Figure 9B. However, this pattern of spatial consistency was not present in the sham group.

To examine the differences between brain regions, the left (right) dlPFC and frontal pole were assigned as regions of interest, named ROI1, ROI2 and ROI3, as was shown in Figure 9C. The changes in edge number were extracted separately from different ROIs and periods, and submitted to repeated measurements ANOVA for testing the differences in cortical plasticity. There was a significant main effect for the follow-up [*F*(2, 46) = 4.36, *p* = 0.019], but not for posttest period [*F*(2, 46) = 2.62, *p* = 0.084]. Further testing showed that ROI1 was significantly greater than ROI2 in posttest (*t* = 2.34, *p* = 0.028) and follow-up (*t* = 2.35, *p* = 0.028); ROI3 is significantly greater than ROI2 (*t* = 2.81, *p* = 0.009), results were shown in Figure 9D.

## Discussion

At the end of the experiment, both the subjects and the experimenter were asked by questionnaire whether they knew the experimental grouping, and they all answered no. On the basis of double blinding and excluding the placebo effect, we propose that fNIRS-based intermittent neurofeedback training directly perturbs the resting-state brain network and improves cognitive performance. Intermittent feedback allows subjects sufficient time to verify their assumptions in a scenario similar to the resting state, and no-feedback self-regulation sessions interspersed in the training protocol may serve as a reinforcement for the transfer from the training state to the resting state.

Three aspects including task state activation, cognitive performance and resting state brain network were analyzed to examine the effectiveness of training. The results indicated that not only can training have beneficial effects on all three aspects in the short term (1 day after training), but the effects can still be observed 1 week after training. For example, the activation of the Stroop task in fNIRS-NF group is still significantly reduced compared to the sham group after 1 week, indicating that training can lead to a long-term decrease in cognitive load. More importantly, suppressing impulsive responses and Stroop conflict effects were believed to be associated with IC (Diamond, 2020; Oldrati et al., 2016), which provides causal inference evidence about inhibitory control and small-world networks.

The brain is an interconnected whole, advanced cognitive functions activate the brain in the form of networks (Erika-Florence et al., 2014). Although fNIRS-NF training based on the average signal a single brain region is an effective method (Hou et al., 2021a; Nouchi et al., 2022), the regulation of global networks may be more exciting because circuits create networks by stringing together many brain regions to orchestrate a brain symphony (de Schotten and Forkel, 2022). Understanding feedback signals is an important process, but it may be covert (Ramot et al., 2016; Ramot and Martin, 2022). An obvious advantage of network-based feedback is that it is more difficult for individuals to understand the meaning of the feedback signal, thus avoiding conscious manipulation of scores but still achieving good results. For example, latest research has used fMRI neurofeedback to sculpt the brain without the participants’ explicit awareness (Iordan et al., 2024).

This also provided a new perspective on inhibitory control. Previous study has demonstrated that enhancing prefrontal small-worldness through feedback training could improve inhibitory control, which is the result of regulating a brain network from randomness to regularity (Zeng et al., 2023). Similarly, in this study, cognitive improvement was accompanied by network regularization, but it seems to contradict the brain network regularization of ADHD patients (Cao et al., 2014; Cao et al., 2013; Wang et al., 2009). Actually, they may be two sides of the same coin. Like the “heart rate reserves” of professional athletes, long-term exercise training results in a decrease in the resting heart rate (Grace et al., 2018), which is not the same as the inherently low resting heart rate shown by antisocial personalities (Portnoy and Farrington, 2015); the former is a resource reserve, and the latter is a clinical pathology. In this study, an increase in small-worldness increased resting-state brain network regularity, which reduces basal consumption while expanding the dynamic of the brain network.

As is well known, right inferior frontal gyrus and dlPFC were crucial brain region for inhibitory control (Hampshire et al., 2010; Angius et al., 2019), but extended cortical regions included in PFC were used for regulation in this study. Some reasons can be used to explain this setting of this experiment: brain regions involved in inhibitory control may be dedifferentiated (Rajah and D’Esposito, 2005; Rieck et al., 2021) and compensatory (Cabeza, 2002; Goh, 2011; Hartwigsen, 2018; Rajah and D’Esposito, 2005; Reuter-Lorenz et al., 2000; Reuter-Lorenz and Park, 2014). Owing to the top-down regulatory role of the frontal cortex in the posterior brain region, the wider recruitment of the PFC by the regularized network makes this cognitive control scheme more precise and efficient, enhancing the selective and specific responses to stimuli (Gazzaley et al., 2008; Gazzaley et al., 2005). The idea that inhibitory control is solely determined by the dorsolateral PFC is not rigorous (Anderson, 2016; Anderson and Penner-Wilger, 2013). Higher brain functions in humans, such as perception, learning and goal-directed behaviors, are often hypothesized to depend on the collective dynamics of many interacting neurons distributed throughout the cortex.

However, typical signs of cooperative phenomena are not accessible through single-neuron investigations (Chialvo, 2010). The entire PFC plays an important role in inhibitory control, and individuals may recruit different brain regions and neurons to perform the same task in a nonlinear, flexible self-organizing manner; this can result in an unexpected collective spatiotemporal pattern, which is a typical emergent phenomenon.

Brain network visualization shows that the brain is recruiting resources from relatively isolated brain regions at the periphery by establishing long-distance connections, which allow for broader interactions between nodes, however, this is a self-organizing behavior without central control. This is similar to the way that ants in colonies are forced to follow their own pheromone trails and explore every direction yet show collective intelligence for foraging behaviors (Fromm, 2005). And the latest research from Stanford University found that when ants work in groups, their performances rise significantly, which is known as collective cognition (Dreyer et al., 2024). The brain and ant colony have similar characteristics: they consist of simple elements (ants or neurons), each of which has simple behavior but exhibit complex and unexpected spatiotemporal patterns when they form a whole through nonlinear interactions.

In addition, visualization of brain network and comparison between ROI cortices provided us with some preliminary insights about cortical plasticity. We found some interesting patterns of node behavior: the left region (e.g., left-dlPFC) is more likely to reduce connections, whereas the marginal region on the right (e.g., right-dlPFC) is more likely to increase connections. This suggested that the connections change asymmetrically, and there are differences in the plasticity of cortical areas. This may be related to the intrinsic structure of the brain and provides targets for future functional connectivity interventions.

Some limitations need to be noted. Firstly, the study did not include patients with IC defects, such as ADHD and autism spectrum disorder, and therefore cannot be directly applied to clinical practice. In addition, the 5 training sessions did not seem to have reached the maximum limit for improvement, and the intervention time can be further extended. Finally, given the diversity of brain network topology properties and cognitive functions, we cannot guarantee that this method is optimal for improving inhibitory control, and further study is needed to elucidate the many-to-many relationship between brain network topology and cognitive function.

## Conclusion

Intermittent network-based fNIRS neurofeedback training is an effective method for regulating resting state brain networks, which is different from traditional neurofeedback based on modular paradigms. Although the network signals are difficult to understand, they are still effective. Using the small world brain network as a feedback target can improve inhibitory control and reduce cognitive load, and the training effect can be lasted for more than one week. Although feedback algorithms do not target specific nodes, cortical regions exhibit different responses: the connections of the right dlPFC increase while those of the left dlPFC decrease, indicating differences in brain plasticity between these regions. Overall, this study provides potential targets for future neurofeedback interventions and offers additional insights into inhibitory control.

## Data Availability

The datasets analyzed during the current study are available from the corresponding authors upon reasonable request.
